# Clinical and epidemiological characteristics of herpes zoster at a tertiary care center compared to population-based data: a ten-year retrospective cohort study

**DOI:** 10.1186/s12879-026-14185-7

**Published:** 2026-08-31

**Authors:** Johanna Lenz, Hans Helmut Niller, Sebastian Haferkamp

**Affiliations:** 1 Institute of Medical Microbiology and Hygien, University of Regensburg, Franz-Josef-Strauß-Allee 11, D-93053 Regensburg, Germany; 2 Department of Dermatology and Venereology, Martin Luther University Halle-Wittenberg, Ernst-Grube-Str. 40, D-06120 Halle (Saale), Germany

**Keywords:** Herpes zoster, Varicella-zoster virus, Tertiary care center, Retrospective cohort study, Postherpetic neuralgia, Herpes zoster ophthalmicus, Immunocompromised, Inpatient treatment

## Abstract

**Background:**

Herpes zoster (HZ) results from reactivation of the latent varicella-zoster virus (VZV) and may cause severe pain and serious complications. The risk increases with age, immunosuppression, and certain chronic conditions. Tertiary care centers disproportionately treat patients with such risk profiles, yet data comparing tertiary care populations with population-based cohort studies are limited. This study aimed to provide a comprehensive clinical and epidemiological characterization of HZ patients at a tertiary care center and to compare findings with those from population-based studies.

**Methods:**

All patients at the University Hospital Regensburg with an HZ diagnosis (ICD code B02*) between January 2014 and December 2023 were included and subsequently underwent manual chart review for clinical validation and data extraction, yielding 786 cases in 780 patients. We analyzed patient demographics, comorbidities, immunosuppression, anatomical location, complications, hospitalization status, and vaccination status.

**Results:**

Of 786 cases, 398 occurred in women and 388 in men, with a median age of 60 years. A total of 13.5% of cases were classified as immunocompromised. The trigeminal nerve was involved in 50.1% of cases, and herpes zoster ophthalmicus (HZO) was present in 41.3%. Most cases, 72.8%, proceeded without complications, with postherpetic neuralgia (PHN) being the most common complication, affecting 8.4% of cases. The hospitalization rate was 57.6%, and HZ vaccination status was documented in fewer than 1% of cases.

**Conclusions:**

Compared to published population-based studies, this cohort showed an increased risk profile for severe disease course as immunocompromised patients and cranial manifestations were markedly overrepresented. Despite that, the complication rate was comparable. Early intravenous antiviral therapy during hospitalization may have contributed to this, though retrospective design precludes causal inference. Prospective studies are needed to confirm this hypothesis. The near-total absence of vaccination records indicates a significant documentation gap and underscores the need for systematic vaccination counselling and documentation in tertiary care centers.

**Supplementary Information:**

The online version contains supplementary material available at https://doi.org/10.1186/s12879-026-14185-7.

## Introduction

### Background

The varicella-zoster virus (VZV) is responsible for chickenpox during the primary infection, usually affecting children [1], and subsequently remains dormant within the host. The reactivation appears as herpes zoster (HZ) and occurs due to a weakened cellular immune response resulting from aging, cancer, immunosuppressive therapies, or particular comorbidities like cardiovascular disease, chronic renal disease, and chronic obstructive pulmonary disease (COPD) [1, 2]. While reactivation can occur following both natural varicella infection and VZV vaccination, the risk is considerably greater following natural infection [3]. In Germany, more than 95% of adults are seropositive for VZV, reflecting a significant vulnerability to HZ [4].

### Clinical presentation

HZ is clinically characterized by a painful, vesicular rash confined to one or few contiguous dermatomes [5], with the rash typically resolving within 7–10 days, though pain generally persists for about one month [6]. Complications include post-herpetic neuralgia (PHN), defined as pain persisting beyond three months, as well as auditory, ocular, meningeal, and visceral involvement [7]. PHN is particularly common in older patients, immunocompromised individuals, and those with depression [8].

### Prevention options and treatment

The recombinant subunit vaccine Shingrix (GSK) has been approved in the European Union since 2018 and is recommended by the German Standing Committee on Vaccination (STIKO) for individuals aged ≥ 60 years [9] and for those aged ≥ 18 years with relevant comorbidities or immunodeficiencies, administered in two doses at 2–6 month intervals [10]. Antiviral therapy with e.g. acyclovir is often recommended to alleviate persistent pain; while oral administration is usually sufficient, intravenous therapy is indicated in high-risk patients, particularly immunocompromised individuals [1]. Patients with complicated courses are often referred to tertiary care facilities, as outpatient settings typically lack the capability for inpatient treatment.

### Epidemiology

At least one in three people will develop HZ during their lifetime, with incidence and complication rates increasing sharply with age [11]. In Germany, prevalence ranges from 5.8 to 9.6 cases per 1,000 person-years [12–14], with a rising global trend suggesting a growing disease burden [15, 16]. As HZ is not notifiable, systematic epidemiological data in Germany remain limited [17].

Tertiary care centers disproportionately treat patients with elevated risk profiles for severe HZ, yet most epidemiological investigations rely on health insurance records, with few studies focusing exclusively on these settings. We therefore aimed to characterize HZ patients clinically and epidemiologically at a tertiary care center and to contextualize findings in relation to population-based studies.

## Methods

### Study design and setting

This retrospective cohort study involved patients of the University Hospital Regensburg (UKR) from January 1, 2014, to December 31, 2023.

### Patient population and eligibility criteria

The inclusion criteria for this study were met by all patients with an ICD diagnosis code B02* (*0, 1, 2, 3, 7, 8, 9) in the digital hospital record who had a diagnosis between January 1, 2014 and December 31, 2023. Each case subsequently underwent manual chart review to confirm the diagnosis and extract clinical data. Moreover, each diagnosis was treated as an independent event because recurrent episodes may present with different risk profiles. Our analysis is based on 786 cases from 780 patients.

### Data collection and variables

A comprehensive analysis was conducted of following diseases that are known to be risk factors: lupus erythematosus, rheumatoid arthritis, chronic obstructive pulmonary disease, cardiovascular conditions, inflammatory bowel disorder, chronic renal disease, (allergic) asthma, diabetes mellitus, depression. Although not diseases in the strict sense, physical trauma and smoking were included in “comorbidities”, as established and potential risk factors [2]. In addition, some of the most common chronic diseases in Germany were examined: thyroid disorders, arterial hypertension, adiposity.

To better understand the impact of the SARS-CoV-2 pandemic on our patient population, a thorough review of all available information regarding the virus was conducted. This included known vaccination, current COVID-19 infection, ongoing COVID-19-related pneumonia, negative test result, and COVID-19 history. Furthermore, all records regarding the vaccination status for HZ were collected.

Possible immunosuppression through HIV/AIDS or immunosuppressive drugs was determined. Additionally, an attempt was made to comprehensively assess the tumor history and present status, as far as traceable by anamnesis. We also examined the HZ complications while mapping the corresponding spinal nerve origins of the affected dermatomes. The treatment setting (inpatient/outpatient) was recorded for each case.

### Statistical analysis

We used descriptive statistics to summarize the study data. The mean value and standard deviation were used for continuous variables and percentages for qualitative ones.

To assess the normality of the patient’s age, we used the Shapiro-Wilk test. Because the ages were not normally distributed, we employed nonparametric tests.

The Mann–Whitney U test was used to compare continuous variables between two groups, and comparisons involving multiple groups were conducted using the Kruskal-Wallis test. The chi-square test was employed to compare categorical variables, while Fisher’s exact test was utilized when the expected cell frequency was less than five. Correlation analyses were determined using Spearman’s correlation coefficient.

The threshold for significance was set at *p* < 0.05. We decided against alpha error correction because this was a purely exploratory analysis. The study results should be interpreted accordingly.

Microsoft^®^ Excel^®^ for Microsoft 365 MSO (Version 2508 Build 16.0.19127.20532) was utilized for data collection, and JASP (Version 0.96) was employed for statistical analyses.

### Use of artificial intelligence tools

Large language models (Claude, Anthropic; DeepL Write; SciSpace Paraphraser) were used to assist with the refinement of the manuscript text. In addition, AI-powered tools were used to support literature research (Claude, Anthropic; SciSpace), to assist with data preparation and statistical programming in JASP and Microsoft Excel (Claude, Anthropic). All content was critically reviewed, verified, and approved by all authors.

## Results

### Temporal trends

From January 1, 2014, to December 31, 2023, a total of 786 cases of HZ were diagnosed at the UKR. Two opposing trends were identified in the annual distribution. The first trend was a gradual decrease in the number of cases between 2014 and 2018, and the second trend was a strong increase after 2018. The highest number of diagnoses occurred in 2014 and 2023, while the lowest number of cases was recorded in 2018. The temporal trend is evident in both the number of inpatients and the number of cases with PHN, although the difference is less pronounced in the latter group.

Although the annual number of cases showed considerable variation, a correlation test over the 10 year period showed no significant link (*r* = 0.248, *p* = 0.492, *n* = 10).

The annual distribution of cases, hospitalized cases, and PHN over the study period is shown in Additional file 1.

When examining seasonal trends, a statistically significant deviation of the number of cases across the months was detected (χ²(11) = 22.031, *p* = 0.024). Patient enrollment data showed two peaks, with the fewest cases in April and October and the most in June and December. The monthly distribution of cases is presented in Additional file 2.

### Patient characteristics

#### Gender, age

We identified 398 females and 388 males, indicating an even gender distribution (χ²(1) = 0.127, *p* = 0.721). The median age of the patients was 60 years, with no significant difference between the sexes (U = 75061, *p* = 0.499). When categorized into age groups of 10 years each, the largest group, comprising 20.6% of all diagnoses, was the 60–69-year-old demographic. The 70–79 and 50–59 age groups were slightly less frequent (16.7% and 16.5%). The age groups with the fewest cases were those aged ≤ 19 and ≥ 90 years (Table 1).

The patients’ age did not correlate with the year of diagnosis (r(784) = 0.017, *p* = 0.631, *N* = 786), indicating a constant age distribution throughout the entire observation period.

**Table 1:** Age group distribution of HZ cases stratified by sex, inpatient admission and PHN

Age Group (in years)	Total (*n* = 786)		Female (*n* = 398)		Male (*n* = 388)		Admission (*n* = 453)		PHN^a^ (*n* = 57)	
	*n*	(%)	*n*	(%)	*n*	(%)	*n*	(%)	*n*	(%)
1–19	25	(3.2)	17	(68.0)	8	(32.0)	16	(64.0)	0	(0.0)
20–29	53	(6.8)	23	(43.4)	30	(56.6)	17	(32.1)	1	(1.9)
30–39	82	(10.5)	37	(45.1)	45	(54.9)	32	(39.0)	2	(2.4)
40–49	89	(11.4)	43	(48.3)	46	(51.7)	45	(50.6)	2	(2.2)
50–59	129	(16.5)	70	(54.3)	59	(45.7)	72	(55.8)	13	(10.1)
60–69	161	(20.6)	81	(50.3)	80	(49.7)	88	(54.7)	17	(10.6)
70–79	130	(16.7)	60	(46.2)	70	(53.8)	90	(69.2)	11	(8.5)
80–89	103	(13.2)	57	(55.3)	46	(44.7)	80	(77.7)	10	(9.7)
>= 90	13	(1.7)	10	(76.9)	4	(30.8)	13	(100.0)	1	(7.7)

Absolute number of cases (n), proportion of total cases per age group (%)

^a^ Postherpetic Neuralgia

### Comorbidities, immunosuppression

In nearly half of the cases (46.1%), none of the comorbidities we identified had been previously diagnosed. Furthermore, 54.7% of the cases did not exhibit any risk factors for HZ reactivation, including immunosuppression and malignancies [2]. The most prevalent comorbidities associated with an elevated risk of HZ were cardiovascular conditions and chronic renal diseases, affecting over 10% of cases in each category (Table 2).

Patients with more comorbidities were also significantly older (*r* = 0.39, *p* < 0.001). While the total number of comorbidities did not differ significantly between the sexes (U = 79758, *p* = 0.39), males showed a higher prevalence of relevant comorbidities, including current malignancies (U = 83351, *p* = 0.026). However, both male and female subjects demonstrated an average of less than one comorbidity (0.64 and 0.50), indicating limited clinical relevance. The male population exhibited a higher prevalence of chronic obstructive pulmonary disease, cardiovascular conditions, and chronic renal diseases. These conditions have been demonstrated to elevate the risk of developing HZ [2]. However, thyroid disorders manifested at a significantly higher frequency among the female population, though these conditions have not been identified as risk factors [2].

Most patients (86.5%) were immunocompetent. However, 7.7% of cases had active malignancies, thus accounting for just over half of immunocompromised cases. The remainder were on immunosuppressive drugs or had HIV/AIDS (Table 2).

**Table 2:** Distribution of co-morbidities and immunosuppression stratified by gender

	Total (*n* = 786)		Female (*n* = 398)		Male (*n* = 388)	
	*n*	(%)	*n*	(%)	*n*	(%)
Co-morbidities						
No Co-morbidities^a^	362	(45.4)	187	(47.0)	175	(45.1)
Smoking^a^	67	(8.5)	26	(6.5)	41	(10.6)
Thyroidal Disease	119	(15.1)	83	(20.9)	36	(9.3)
Hypertension	220	(28.0)	106	(26.6)	114	(29.4)
Adipositas	43	(5.5)	21	(5.3)	22	(5.7)
One or more Relevant Co-morbidities^b, c^	279	(35.5)	129	(32.4)	150	(38.7)
Lupus Erythematosus (LE)	2	(0.3)	1	(0.3)	1	(0.3)
Rheumatoid Arthritis (RA)	18	(2.3)	12	(3.0)	6	(1.5)
Chronic Obstructive Pulmonary Disease (COPD)	22	(2.8)	6	(1.5)	16	(4.1)
Cardiovascular Conditions	125	(15.9)	53	(13.3)	72	(18.6)
Inflammatory Bowel Disease (IBD)	10	(1.3)	4	(1.0)	6	(1.5)
Chronic Renal Disease	90	(11.5)	31	(7.8)	59	(15.2)
Asthma	16	(2.0)	9	(2.3)	7	(1.8)
Diabetes	82	(10.4)	42	(10.6)	40	(10.3)
Depression	34	(4.3)	20	(5.0)	14	(3.6)
Physical Trauma^b^	7	(0.9)	2	(0.5)	5	(1.3)
**Immunosuppression**						
Without immunosuppression^c^	680	(86.5)	362	(91.0)	338	(87.1)
Immunosuppressive drugs	44	(5.6)	18	(4.5)	26	(6.7)
HIV/AIDS	2	(0.3)	0	(0.0)	2	(0.5)
Current Malignancies	60	(7.6)	18	(4.5)	22	(5.7)
Chemotherapy^d^	27	(3.4)	11	(2.8)	16	(4.1)
Radiotherapy^d^	8	(1.0)	2	(0.5)	6	(1.5)
Surgical Therapy^d^	12	(1.5)	6	(1.5)	6	(1.5)
Malignancy History^e^	70	(8.9)	35	(8.8)	35	(9.0)

Absolute number of cases (n), proportion of total cases within each group (%). Percentages may not sum to 100% due to multiple co-morbidities per patient

^a^ Although not a comorbidity in the strict sense, smoking was included as common habit with negative health – impact

^b^ Although not a comorbidity in the strict sense, physical trauma was included as established risk factor (2)

^c^ Defined as HIV/AIDS or current Malignancies(2) or intake of immunosuppressive drugs

^d^ Current or planned treatment. Multiple treatment options may be pursued simultaneously. Percentages may not sum to 100% due to lack of data

^e^ Malignancies that have undergone complete treatment

Abbreviations: HIV, human immunodeficiency virus

### SARS-CoV-2, zoster vaccination

COVID-19-related data were available for 49 of 226 cases documented during the peak pandemic period in Bavaria between March 2020 and April 2023 [18], with 59.2% providing only a negative SARS-CoV-2 test result. Given this limited data coverage, a conclusive association between the pandemic and the incidence of HZ could not be determined. Available SARS-CoV-2 data are summarized in Additional file 3.

HZ vaccination status was documented in only three cases, precluding any representative analysis.

### Anatomic location

In our cohort the most common manifestations (67.6%) involved the cranial and cervical ganglia. The trigeminal nerve was affected in half of all cases (50.1%), while the ophthalmic nerve branch was involved in 82.5% of these cases.

Approximately one-fifth of cases developed HZ in the thoracic dermatomes. Table 3 presents the exact distribution.

A significant difference in median age was observed across anatomic locations (H(7) = 20.58, *p* = 0.004, *N* = 786). Patients with HZ reactivation in the cervical segments and trigeminal involvement were the oldest (64 and 63 years), while the youngest patients had affected thoracic dermatomes and otic nerves (55 and 54 years).

Cases with affected otic nerve had the highest incidence of complications (75.4%), while it was second highest for affected trigeminal nerve (28.4%). There was almost no difference when the ophthalmic branch was involved (28.9%).

**Table 3:** Anatomic location of HZ manifestation and related inpatient admissions

Anatomic location	Total (*n* = 786)		Admissions (*n* = 453)	
	*n*	(%^a^)	*n*	(%^b^)
Trigeminal nerve	394	(50.1)	268	(68.0)
Ophthalmic nerve	325	(41.3)	224	(68.9)
Maxillary nerve	73	(9.3)	50	(68.5)
Mandibular nerve	20	(2.5)	13	(65.0)
Otic nerve	65	(8.3)	53	(81.5)
Cervical segments	72	(9.2)	31	(43.1)
Thoracic segments	143	(18.2)	60	(42.0)
Lumbar segments	53	(6.7)	17	(32.1)
Sacral segments	24	(3.1)	7	(29.2)
Multisegmental	6	(0.8)	4	(66.7)
Unspecified	29	(3.7)	12	(41.4)

Absolute number of cases (n), proportion of total cases (%^a^) and proportion of admissions per cases with same anatomic location (%^b^). Percentages may not sum to 100% due to disseminated HZ

### Complications

Most HZ cases proceeded without complications (72.8%). The most common complication was PHN, which affected 8.4% of cases. No significant differences were found between the sexes, but more than 90% of cases with PHN were ≥ 50 years old. The prevalence was 9.7% in patients aged ≥ 50 years, but it did not increase in older age groups.

Other complications that occurred approximately half the rate of PHN included intraocular complications (3.4%) and superinfection (4.2%). Encephalitis, meningitis, and necrotizing HZ were the least common complications. Table 4 shows the exact incidence of all complications.

A significant association was found between the number of comorbidities and the number of complications (*r* = 0.096, *p* = 0.007), as well as when relevant comorbidities, including current malignancy, were considered (*r* = 0.076, *p* = 0.034). However, both correlations represented negligible effect sizes (*r* < 0.1), suggesting limited clinical relevance.

Complications affected men and women with equal frequency (χ² = 0.437, *p* = 0.508), and no association was found between sex and the occurrence of individual complications. Patients who did not experience complications were significantly younger than those who did (U = 71161, *p* < 0.001). The patient population was significantly older for certain complications, such as PHN (U = 18580, *p* = 0.003), zoster keratitis (U = 6330, *p* = 0.01), and intraocular complications (U = 9671, *p* = 0.016).

**Table 4:** Complications that occurred in HZ cases stratified by sex

Complications	Total (*n* = 786)		Female (*n* = 398)		Male (*n* = 388)	
	*n*	(%)	*n*	(%)	*n*	(%)
No complications	572	(72.8)	285	(71.6)	287	(74.0)
Postherpetic Neuralgia (PHN)	66	(8.4)	42	(10.6)	24	(6.2)
Intraocular Complications	34	(4.3)	13	(3.3)	21	(5.4)
Superinfection	33	(4.2)	17	(4.3)	16	(4.1)
Inner Ear Involvement	26	(3.3)	18	(4.5)	8	(2.1)
Herpes Zoster Keratitis	24	(3.1)	14	(3.5)	10	(2.6)
Herpes Zoster Generalisatus	20	(2.5)	7	(1.8)	13	(3.4)
Encephalitis or Meningitis	16	(2.0)	8	(2.0)	8	(2.1)
Necrotizing Zoster	10	(1.3)	3	(0.8)	7	(1.8)

Absolute number of cases (n), proportion of total cases within each group (%). Percentages may not sum to 100% due to multiple complications per patient

### Inpatient stays

Of all HZ cases, more than half (57.6%) were treated as inpatients, with age and anatomical location playing a significant role. Most admissions were due to zoster affecting the trigeminal nerve, followed by the thoracic segments and otic nerve.

The median age of the inpatients was 65.0 years. Patients were admitted to the hospital more frequently as they advanced in age. Only one-third (32.1%) of cases aged 20–29 years were admitted; however, this percentage steadily increased in older age groups, reaching a peak among patients aged ≥ 90 years. The only exception to this pattern was among 1-19-year-olds, of whom nearly two-thirds (64.0%) were also treated as inpatients (Table 1). The median age of women and men undergoing inpatient treatment did not differ significantly (U = 24191, *p* = 0.325). The incidence of hospitalization for HZ was highest in patients with an affected otic nerve (81.5%). Moreover, most cases with trigeminal involvement were also treated as inpatients (68.0%). The lowest admission rate was seen in patients with HZ in the lumbar and sacral dermatomes (Table 3).

## Discussion

This study identified a cohort at elevated risk for HZ complications, characterized by advanced age, a high prevalence of immunosuppression, and marked overrepresentation of cranial manifestations. Despite this risk profile, complication rates were comparable to those reported in population-based studies.

### Patient characteristics

#### Gender and age

An equitable distribution of gender was observed within the study cohort (398 females and 388 males). This observation does not align with findings from analogous literature. A comprehensive analysis of all hospitals across Germany, including the UKR, reported 58% of cases in females, which was significantly more than the proportion of affected males [15]. Additionally, female sex has been identified as a risk factor for the onset of this disease [19].

Our findings revealed a similar clinical presentation in males and females. Sex did not affect the progression of the disease. Neither risk factors such as age, relevant comorbidities, and immunosuppression, nor the course of disease characterized by anatomical distribution, hospitalization rates and complications differed significantly. The median age of patients enrolled in the study was 60 years. Accordingly, our cohort comprised an increased ratio of older patients relative to population-centered research, in which the average age ranged from 51 to 52 years, or the median age was recognized as 56 years [20–22]. However, approximately two-thirds (68%) of the cases were 50 years of age or older, a finding supported by existing literature regarding HZ in the German population [13]. On the other hand, a comparable monocentric study at a German university hospital reported an even higher median age of 64 years [23]. This suggests that monocentric studies focusing on tertiary care facilities may encompass a higher proportion of older patients than is typically observed in population-based research. This is particularly significant, given the elevated risk of complications and associated disease progression that correlates with increasing age [8]. Furthermore, older patients are more likely to experience persistent pain, such as PHN [24].

#### Comorbidities and immunosuppression

Just over half of the cases (54.6%) were found to have at least one comorbidity. Particular comorbidities, such as cardiovascular conditions and chronic renal diseases, are acknowledged for their potential to increase the risk of HZ reactivation [2]. These comorbidities are most likely to be more prevalent among individuals receiving treatment at a tertiary medical institution than in the general population. Thus, investigations involving older demographics or exclusively inpatient populations reported a higher prevalence of such patients compared to our findings, ranging from 62.7% to 73.8% [14, 25], whereas the percentages in population-based analysis were notably lower, falling between 28.1% and 30% [24, 26]. Immunosuppression significantly influences the occurrence of HZ, affecting both the overall likelihood of disease manifestation and the frequency of associated complications [27, 28]. In our investigation, 13.5% of cases were classified as immunosuppressed, meaning they were either undergoing treatment with immunosuppressive agents, had a pre-existing malignancy, or were positive for HIV. These data illustrate a considerably higher prevalence than what has been reported in population-based studies, which suggest ratios between 2.8% and 6.6% [21, 26]. It is important to acknowledge that the precise definition of immunosuppression varies slightly across different studies. While a direct comparison of statistics is therefore not possible, the elevated proportion of patients with compromised immune systems remains evident when juxtaposed with existing literature.

#### Anatomical distribution

In the present investigation, the reactivation of HZ at the trigeminal nerve was observed in 50.1% of the subjects, establishing it as the predominant anatomical site. Thoracic dermatomes were involved in 18.2% of cases. This distribution appears inversely represented compared to published literature, which consistently reports trigeminal involvement at approximately 15% and thoracic involvement at 53–55% of cases [29, 30].

One possible explanation for this discrepancy could be found in the advanced age of the participants in our investigation, as the prevalence of thoracic lesions diminishes with age, whereas trigeminal involvement tends to increase [31]. Within the trigeminal nerve cases, a further notable finding emerged: in 82.5% of these cases, the ophthalmic branch was affected, a condition also referred to as herpes zoster ophthalmicus (HZO). Systematic literature reviews have documented HZO incidence ranging from 1.4% to 15.9% [8, 11]. Our study found a notably higher incidence of 41.3%. Another study examining HZO patients at a German clinic with an eye center reported HZO incidence rates ranging from approximately 30% to 60% over a period of more than 10 years [23]. This indicates selection bias in hospitals with ocular departments, as HZO patients should be treated as inpatients and undergo an ophthalmological examination to determine ocular involvement [1].

HZO can cause a range of ocular complications, such as keratitis, uveitis, and conjunctivitis. These complications have been reported to occur in 30% to 78% of HZO patients [11, 32]. We found ocular complications in 18.2% of HZO patients, indicating a notably low complication rate. Studies examining population-based cohorts described incidences for these complications ranging from 0.0% to 4.4% [8], whereas in our analyses, they occurred in 7.5% of all cases. Considering that our cohort had more than two and a half times the number of cases with HZO than population-based studies (41.3% vs. ≤15.9%), this is a relatively low proportion of ocular complications. Furthermore, elevated complication rates in HZO patients have been observed in patients with particular forms of immunosuppression, including those with HIV/AIDS and recipients of stem cell transplants [8]. This indicates an even higher risk for HZO-related complications in our cohort, as 13.5% of all cases were immunosuppressed.

#### Complications

Nearly three quarters (72.8%) of cases with HZ proceeded without complications. This percentage aligns closely with population-based health insurance data from Germany (72.4% − 77.5%) [33, 34] and is consistent with the global literature (67.1% − 86.2%) [8]. However, among inpatient cases only, 36.4% experienced no complications. A study conducted in tertiary care centers in a specific region of Italy (35) reported a remarkably similar figure (36.2%), while 74.4% of cases were complication-free in Chinese healthcare facilities [25].

Patients who experienced complications were statistically significantly older than those who did not. Other studies have likewise demonstrated an increased occurrence of complications with older age [28, 35]. The likelihood of complications is also elevated under conditions of immunosuppression [28].

The most prevalent complication was PHN, which developed in a total of 8.4% of cases. PHN is commonly defined by clinically significant pain persisting for more than 90 days following the onset of the rash [11]. While the incidence rate was recorded at only 4.5% in a study examining German health insurance data [13], global systematic reviews report a range of 2.6% to 46.7% within population-based cohorts [8, 11]. PHN was found to be more prevalent in patients with immunosuppression and certain comorbidities, such as diabetes and depression, compared to immunocompetent individuals [8]. Older patients also demonstrated a higher frequency of occurrence. The variance in reported rates was partly due to inconsistent definitions of PHN, which can differ in terms of pain duration or intensity. Additionally, retrospective studies tended to report lower incidence rates than prospective studies [11].

The association of PHN with age is well-documented [8], and this study clearly corroborated that connection. A substantial 91.5% of cases with PHN were 50 years of age or older, indicating a prevalence of 9.7% among individuals aged 50 and above. A similar figure (11.9%) was observed in a study involving primary care centers in Germany [14]. However, it is noteworthy that in our study, the risk of developing PHN did not increase further with advancing age. Even among those aged 70 years and above, only 8.9% were affected, whereas other studies reported a continuous increase in risk, particularly among the elderly population [13, 14, 24].

Another significant risk factor for PHN is immunosuppression. Depending on the study and the degree of immunosuppression, it has been reported to be slightly elevated to twice as high [36, 37]. Additionally, patients diagnosed with HZO are more likely to develop PHN [38]. Beyond their clinical significance, complications such as PHN can have a lasting negative impact on patients’ quality of life [39, 40].

#### Inpatient admission

In the current study, 57.6% of the cohort was admitted to the hospital. As expected, this ratio was significantly higher than the maximum of 8% observed in population-based studies [7, 28, 33].

The presence of immunosuppression, along with the specific anatomical site of zoster reactivation, significantly influences the decision to initiate inpatient treatment. When the manifestation occurs within the craniofacial region, inpatient intravenous therapy utilizing acyclovir is imperative [1]. Within our studied cohort, craniofacial involvement affected a substantial majority of cases (67.6%), with a notably elevated percentage (13.5%) being classified as immunosuppressed. This observation elucidates the elevated rate of hospitalization.

Furthermore, a positive association between the frequency of inpatient treatment and advancing age was observed. To be precise, the admission rate was 32.1% for individuals aged 20–29, doubling to 77.7% for 80–89-year-olds. Existing literature corroborates this phenomenon [15, 28], with additional studies focusing exclusively on inpatient cohorts also documenting a high mean age ranging from 63 to 64 years [25, 41].

Beyond patient characteristics, the treatment modality itself may be relevant. Inpatient treatment enabled timely intravenous antiviral therapy, as opposed to oral pharmacotherapy in outpatient settings. Whether this contributed to the comparatively low complication rate observed in this cohort remains speculative; however, the retrospective design precludes any causal inference regarding treatment effects. Prospective studies comparing inpatients and outpatients with similar risk profiles and anatomical locations of HZ lesions should be conducted at tertiary care centers to evaluate this hypothesis.

#### Temporal trend

Two opposing trends were identified over the period of ten years. There was a substantial reduction in cases from 2014 to 2018 exceeding 80%, followed by a sevenfold increase from 2018 to 2023. However, no statistically significant correlation was found between patient numbers and diagnosis year (*r* = 0.248, *p* = 0.492, *n* = 10).

Existing literature does not support this V-shaped trend. Instead, it shows a steady annual increase in HZ patients [15, 16] of 3% to 4% per year [22]. The study’s timeline aligns with the COVID-19 pandemic from March 2020 onwards [18]. This pandemic has been previously linked to increased HZ incidence [42]. An increase in cases was noted during the pandemic in our data as well. However, documentation was insufficient for thorough investigation (available information in only 49 of 226 cases).

While an unconventional temporal trend is apparent in our study, definitive conclusions regarding annual trends or the effects of the pandemic cannot be drawn from these data.

Beyond temporal trends, the vaccination status of our cohort warrants attention. While population-level data from a German health insurance provider suggest that vaccination coverage has risen to 15.4% among individuals aged 60 and older in Bavaria between 2019 and 2023, vaccination rates remain critically insufficient [43]. This is particularly striking given that HZ vaccination has been fully reimbursed by statutory health insurance in Germany for individuals aged 60 years and older, and for high-risk groups from the age of 50, since May 2019 [44]. Physician-related and organizational factors at the practice level have nonetheless been identified as key determinants of inadequate vaccination rates [43]. Although vaccination is primarily a task of primary care, the concentration of high-risk patients in tertiary care centers creates a particular responsibility for targeted vaccination counseling. Systematic documentation of vaccination status is a necessary prerequisite for this, and its near-total absence in our cohort suggests that vaccination has not yet been sufficiently prioritized in this setting.

### Strengths and limitations

#### Strengths

All verifiable cases of HZ at the UKR were recorded and evaluated over a 10-year period. This revealed detailed clinical data from the tertiary care setting, thus offering an accurate representation of current clinical practices within a tertiary care facility concerning HZ. The investigation examined and presented not only the most important pre-existing conditions, but also the distribution of the anatomical locations of HZ reactivation and complications in detail.

#### Limitations

This was a single-center, purely retrospective study with a correspondingly limited sample size (*N* = 786). Many patients were referred to the UKR, which created a selection bias and rendered the cohort unrepresentative of the general population. The higher comorbidity burden of patients treated at a tertiary care center compared to population-based cohorts may itself contribute to differences in observed outcomes, independent of treatment modality. As detailed therapy data including timing of antiviral initiation were not systematically collected, the hypothesis regarding the contribution of intravenous antiviral therapy to the observed complication rates cannot be evaluated from the present data. There were deficiencies in documentation, particularly concerning HZ vaccination status (only 3 cases recorded) and COVID-19 status (49 out of 226 cases recorded) during the pandemic. The retrospective data collection was further limited by incomplete documentation of comorbidities and tumor histories, potentially resulting in missed pre-existing conditions and malignancies. Furthermore, there was no systematic follow-up, precluding the interpretation of recurrences and long-term complications. Due to the small number of cases and the short observation period, no valid conclusions could be drawn regarding temporal and seasonal trends or the gender distribution that differed from national hospital data. Since the UKR did not have a fixed area of operation, population-based calculations were not possible. This severely limited direct comparisons with studies that reported incidences in person-years or adjusted for the population. Possible differences in the definitions of certain complications, pre-existing conditions, and immunosuppression must also be considered when comparing this study to others.

## Conclusions

In this study, we analyzed HZ patients from a tertiary care center over the course of ten years, comparing our findings with those from population-based research. We identified a high-risk group within our cohort. These patients were generally older and more often immunosuppressed. They also experienced reactivation in cranial ganglia, especially with ophthalmic involvement, which likely reflects referral patterns and selection bias inherent to a tertiary care center with an ophthalmology department. Despite this, the complication rate was similar to that of published population-based studies.

High rates of inpatient treatment with intravenous antiviral therapy may have contributed to this outcome; however, the retrospective design precludes any causal inference regarding treatment effects. Prospective studies are needed to confirm this hypothesis.

Our findings underscore that high-risk patients who benefit most from vaccination are often treated in tertiary care centers. Vaccination status was documented in only 0.4% of cases, suggesting that vaccination history is neither systematically assessed nor recorded in this setting, which may indicate a missed preventive opportunity. We therefore recommend prioritizing systematic vaccination counseling and documentation to prevent HZ reactivation and its complications.

## Supplementary Information

Below is the link to the electronic supplementary material.


Supplementary Material 1: Additional file 1: Annual distribution of HZ cases, hospitalized cases, and PHN at the University Hospital Regensburg, 2014–2023.



Supplementary Material 2: Additional file 2: Monthly distribution of HZ cases at the University Hospital Regensburg, 2014–2023.



Supplementary Material 3: Additional file 3: Available data regarding SARS-CoV-2.


## Data Availability

The datasets used and analyzed during the current study are available from the corresponding author on reasonable request.

## Abbreviations

COPD: Chronic obstructive pulmonary disease
HZ: Herpes zoster
HZO: Herpes zoster ophthalmicus
PHN: Postherpetic neuralgia
STIKO: Ständige Impfkommission (Standing German Committee on Vaccination)
UKR: Universitätsklinikum Regensburg (University Hospital Regensburg)
VZV: Varicella-zoster virus

## References

[1] Gross GE, Eisert L, Doerr HW, Fickenscher H, Knuf M, Maier P, et al. S2k guideline for the diagnosis and therapy of zoster and post-zoster neuralgia. GMS Infect Dis. 2020;8:Doc01. 32373426 10.3205/id000045 PMC7187398

[2] Marra F, Parhar K, Huang B, Vadlamudi N. Risk Factors for herpes zoster infection: a meta-analysis. Open Forum Infect Dis. 2020;7(1):ofaa005. Available from: URL: https://pmc.ncbi.nlm.nih.gov/articles/PMC6984676/. 10.1093/ofid/ofaa005 PMC6984676 32010734

[3] Civen R, Chaves SS, Jumaan A, Wu H, Mascola L, Gargiullo P, et al. The incidence and clinical characteristics of herpes zoster among children and adolescents after implementation of varicella vaccination. Pediatr Infect Dis J. 2009;28(11):954–9. Available from: URL: https://journals.lww.com/pidj/fulltext/2009/11000/the_incidence_and_clinical_characteristics_of.4.aspx. 10.1097/INF.0b013e3181a90b16 19536039

[4] Robert Koch-Institut. Windpocken (Varizellen), Gürtelrose (Herpes zoster). RKI2024 2026 Jan 27 [cited 2026 Apr 10]. Available from: URL: https://www.rki.de/DE/Aktuelles/Publikationen/RKI-Ratgeber/Ratgeber/Ratgeber_Varizellen.html?nn=16777040#doc16805198bodyText3.

[5] Robert Koch-Institut. Epidemiologisches Bulletin 36/2017. Robert Koch-Institut; 2017. Available from: URL: https://edoc.rki.de/handle/176904/4899.

[6] Drolet M, Brisson M, Levin MJ, Schmader KE, Oxman MN, Johnson RW, et al. A prospective study of the herpes zoster severity of illness. Clin J Pain. 2010;26(8):656–66. 20842005 10.1097/AJP.0b013e3181eef686

[7] Forbes HJ, Bhaskaran K, Grint D, Hu VH, Langan SM, McDonald HI, et al. Incidence of acute complications of herpes zoster among immunocompetent adults in England: a matched cohort study using routine health data. Br J Dermatol. 2021;184(6):1077–84. Available from: URL: https://pmc.ncbi.nlm.nih.gov/articles/PMC8607468/. 10.1111/bjd.19687 PMC8607468 33216946

[8] Giannelos N, Curran D, Nguyen C, Kagia C, Vroom N, Vroling H. The incidence of herpes zoster complications: a systematic literature review. Infect Dis Ther. 2024;13(7):1461–86. Available from: URL: https://pubmed.ncbi.nlm.nih.gov/38896390/. 10.1007/s40121-024-01002-4 PMC11219681 38896390

[9] Siedler A, Koch J, Garbe E, Hengel H, von Kries R, Ledig T et al. Background paper to the decision to recommend the vaccination with the inactivated herpes zoster subunit vaccine. Bundesgesundheitsblatt - Gesundheitsforschung - Gesundheitsschutz. 2019;62(3):352–76. Available from: URL: https://link.springer.com/content/pdf/. 10.1007/s00103-019-02882-5 30848293

[10] Robert Koch-Institut. Beschluss und wissenschaftliche Begründung für die Erweiterung der Indikationsimpfempfehlung zur Herpes-zoster-Impfung mit dem adjuvantierten Subunit-Totimpfstoff für Personen ≥ 18 Jahre mit einem erhöhten Risiko, an Herpes zoster zu erkranken 2025 [cited 2026 Apr 3]; (45/2025). Available from: URL: https://www.rki.de/DE/Aktuelles/Publikationen/Epidemiologisches-Bulletin/2025/45_25.pdf?__blob=publicationFile&v=5.

[11] Kawai K, Gebremeskel BG, Acosta CJ. Systematic review of incidence and complications of herpes zoster: towards a global perspective. BMJ open. 2014;4(6):e004833. Available from: URL: https://pubmed.ncbi.nlm.nih.gov/24916088/. 10.1136/bmjopen-2014-004833 PMC4067812 24916088

[12] Schiffner-Rohe J, Jow S, Lilie HM, Köster I, Schubert I. Herpes zoster in Deutschland. Eine retrospektive Analyse von GKV-Daten. MMW Fortschritte der Medizin. 2010;151 Suppl 4:193–7. Available from: URL: https://pubmed.ncbi.nlm.nih.gov/21595148/. 21595148

[13] Ultsch B, Köster I, Reinhold T, Siedler A, Krause G, Icks A, et al. Epidemiology and cost of herpes zoster and postherpetic neuralgia in Germany. Eur J Health Econ. 2013;14(6):1015–26. Available from: URL: https://pubmed.ncbi.nlm.nih.gov/23271349/. 10.1007/s10198-012-0452-1 23271349

[14] Schmidt-Ott R, Schutter U, Simon J, Nautrup BP, von Krempelhuber A, Gopala K, et al. Incidence and costs of herpes zoster and postherpetic neuralgia in German adults aged ≥ 50 years: a prospective study. J Infect. 2018;76(5):475–82. Available from: URL: https://pubmed.ncbi.nlm.nih.gov/29428228/. 10.1016/j.jinf.2018.02.001 29428228

[15] Zoch-Lesniak B, Tolksdorf K, Siedler A. Trends in herpes zoster epidemiology in Germany based on primary care sentinel surveillance data, 2005–2016. Hum Vaccines Immunotherapeutics. 2018;14(7):1807–14. 10.1080/21645515.2018.1446718 PMC6067859 29498894

[16] van Oorschot D, Vroling H, Bunge E, Diaz-Decaro J, Curran D, Yawn B. A systematic literature review of herpes zoster incidence worldwide. Human Vaccines & Immunotherapeutics. 2021;17(6):1714–32. Available from: URL: https://www.ncbi.nlm.nih.gov/pmc/articles/PMC8115759/. 10.1080/21645515.2020.1847582 PMC8115759 33651654

[17] Robert Koch-Institut. Falldefinitionen des RKI 2026: Gürtelrose (Herpes zoster; Varicella-Zoster-Virus): Robert Koch-Institut; 2026 [cited 2026 Apr 8]. Available from: URL: https://www.rki.de/DE/Themen/Infektionskrankheiten/Meldewesen/Falldefinitionen/Downloads/Guertelrose.pdf?__blob=publicationFile&v=4.

[18] Bayerisches Landesamt für Gesundheit und Lebensmittelsicherheit. 2024. Coronavirus: Übersichtskarte der Fälle in Bayern; 2020 [cited 2026 Mar 26]. Available from: URL: https://www.lgl.bayern.de/gesundheit/infektionsschutz/infektionskrankheiten_a_z/coronavirus/fallzahlen_corona/index.htm.

[19] Steinmann M, Lampe D, Grosser J, Schmidt J, Hohoff ML, Fischer A, et al. Risk factors for herpes zoster infections: a systematic review and meta-analysis unveiling common trends and heterogeneity patterns. Infection. 2024;52(3):1009–26. Available from: URL: https://pubmed.ncbi.nlm.nih.gov/38236326/. 10.1007/s15010-023-02156-y PMC11142967 38236326

[20] Johnson BH, Palmer L, Gatwood J, Lenhart G, Kawai K, Acosta CJ. Annual incidence rates of herpes zoster among an immunocompetent population in the United States. BMC Infect Dis. 2015;15:502. Available from: URL: https://pubmed.ncbi.nlm.nih.gov/26546419/. 10.1186/s12879-015-1262-8 PMC4636742 26546419

[21] Kawai K, Yawn BP, Wollan P, Harpaz R. Increasing incidence of herpes zoster over a 60-year period from a population-based study; 2016 [cited 05.03.26]. Available from: 10.1093/cid/ciw296. PMC4928389 27161774

[22] Thompson RR, Kong CL, Porco TC, Kim E, Ebert CD, Acharya NR. Herpes zoster and postherpetic neuralgia: changing incidence rates from 1994 to 2018 in the United States. Clin Infect Dis. 2021;73(9):e3210-e3217. Available from: URL: https://pmc.ncbi.nlm.nih.gov/articles/PMC8563174/. 10.1093/cid/ciaa1185 PMC8563174 32829399

[23] Diehl R, Wiedenmann C, Reinhard T, Böhringer D, Schauer F. Increasing hospitalisation of patients with herpes zoster ophthalmicus-an interdisciplinary retrospective analysis. Graefes Arch Clin Exp Ophthalmol. 2024;262(2):583–8. 37861849 10.1007/s00417-023-06277-w PMC10844404

[24] Södergren E, Mårdberg K, Nishimwe M, Bhavsar A, Marijam A, Bergström T, et al. Incidence and Burden of herpes zoster in Sweden: a regional population-based register study. Infectious Diseases and Therapy. 2024;13(1):121–40. Available from: URL: https://www.ncbi.nlm.nih.gov/pmc/articles/PMC10828402/. 10.1007/s40121-023-00902-1 PMC10828402 38193987

[25] Chen P, Chen Z, Xiao Y, Chen X, Li J, Tang Y, et al. Characteristics and economic burden of hospitalized patients with herpes zoster in China, before vaccination. Human Vaccines & Immunotherapeutics. 2023;19(3):2268990. Available from: URL: https://www.ncbi.nlm.nih.gov/pmc/articles/PMC10760360/. 10.1080/21645515.2023.2268990 PMC10760360 37899682

[26] Forbes HJ, Bhaskaran K, Thomas SL, Smeeth L, Clayton T, Langan SM. Quantification of risk factors for herpes zoster: population based case-control study. BMJ. 2014;348:g2911. Available from: URL: https://www.bmj.com/content/348/bmj.g2911. 10.1136/bmj.g2911 PMC4019782 25134101

[27] Algaadi SA. Herpes zoster and COVID-19 infection: a coincidence or a causal relationship? Infection. 2022 [cited 2026 Mar 16];50(2):289–93. Available from: URL: https://www.ncbi.nlm.nih.gov/pmc/articles/PMC8607065/. 10.1007/s15010-021-01714-6 PMC8607065 34807403

[28] Buchan SA, Daneman N, Wang J, Garber G, Wormsbecker AE, Wilson SE, et al. Incidence of Hospitalizations and Emergency Department Visits for Herpes Zoster in Immunocompromised and Immunocompetent Adults in Ontario, Canada, 2002–2016. Clin Infect Dis. 2019;71(1):22–9. 10.1093/cid/ciz769 31436814

[29] Pragya A, Nair BC. Patel. Herpes Zoster. In: Nair PA, Patel BC, editors. StatPearls [Internet]. StatPearls Publishing; 2023. Available from: URL: https://www.ncbi.nlm.nih.gov/books/NBK441824/#article-22841.s5.

[30] Sundaram M, Adikrishnan S, Krishnakanth M, Sudha R, Mahalakshmi V, Shobana S, et al. Hospital based cross sectional study of herpes zoster with reference to HIV seropositivity. BMC Infect Dis. 2012;12(Suppl 1):58.

[31] Shiraki K, Toyama N, Shiraki A, Yajima M. Age-dependent trigeminal and female-specific lumbosacral increase in herpes zoster distribution in the elderly. J Dermatol Sci. 2018;90(2):166–71. 29395575 10.1016/j.jdermsci.2018.01.009

[32] Litt J, Cunningham AL, Arnalich-Montiel F, Parikh R. Herpes Zoster Ophthalmicus: Presentation, Complications, Treatment, and Prevention. Infect Dis Therapy. 2024;13(7):1439–59. 10.1007/s40121-024-00990-7 PMC11219696 38834857

[33] Hillebrand K, Bricout H, Schulze-Rath R, Schink T, Garbe E. Incidence of herpes zoster and its complications in Germany, 2005–2009. J Infect. 2015;70(2):178–86. Available from: URL: https://pubmed.ncbi.nlm.nih.gov/25230396/. 10.1016/j.jinf.2014.08.018 25230396

[34] Ultsch B, Siedler A, Rieck T, Reinhold T, Krause G, Wichmann O. Herpes zoster in Germany: quantifying the burden of disease. BMC Infect Dis. 2011;11:173. Available from: URL: https://www.ncbi.nlm.nih.gov/pmc/articles/PMC3141411/. 10.1186/1471-2334-11-173 PMC3141411 21679419

[35] Li Y, An Z, Yin D, Liu Y, Huang Z, Xu J, et al. Disease burden due to herpes zoster among population aged ≥ 50 years old in China: a community based retrospective survey. PLoS ONE. 2016;11(4):e0152660. 27055179 10.1371/journal.pone.0152660 PMC4824529

[36] Bardach AE, Palermo C, Alconada T, Sandoval M, Balan DJ, Nieto Guevara J et al. Herpes zoster epidemiology in Latin America: A systematic review and meta-analysis. PLOS ONE. 2021; 16(8):e0255877. Available from: URL: https://journals.plos.org/plosone/article?id=10.1371/journal.pone.0255877. 10.1371/journal.pone.0255877 PMC8360515 34383851

[37] Forbes HJ, Bhaskaran K, Thomas SL, Smeeth L, Clayton T, Mansfield K, et al. Quantification of risk factors for postherpetic neuralgia in herpes zoster patients: a cohort study. Neurology. 2016;87(1):94–102. Available from: URL: https://www.neurology.org/doi/10.1212/WNL.0000000000002808?url_ver=Z39.88-2003&rfr_id=ori:rid:crossref.org&rfr_dat=cr_pub%20%200pubmed. 10.1212/WNL.0000000000002808 PMC4932239 27287218

[38] Forbes HJ, Thomas SL, Smeeth L, Clayton T, Farmer R, Bhaskaran K, et al. A systematic review and meta-analysis of risk factors for postherpetic neuralgia. Pain. 2016;157(1):30–54. 26218719 10.1097/j.pain.0000000000000307 PMC4685754

[39] Johnson RW, Bouhassira D, Kassianos G, Leplège A, Schmader KE, Weinke T. The impact of herpes zoster and post-herpetic neuralgia on quality-of-life. BMC Med. 2010;8(1):37. 20565946 10.1186/1741-7015-8-37 PMC2905321

[40] Katz J, Cooper EM, Walther RR, Sweeney EW, Dworkin RH. Acute Pain in Herpes Zoster and Its Impact on Health-Related Quality of Life. Clin Infect Dis. 2004;39(3):342–8. 15307000 10.1086/421942

[41] Scampoli P, Di Martino G, Cedrone F, Odio C, Di Giovanni P, Romano F, et al. The burden of herpes zoster on hospital admissions: a retrospective analysis in the years of 2015–2021 from the Abruzzo Region, Italy. Vaccines. 2024;12(5). Available from: URL: https://www.ncbi.nlm.nih.gov/pmc/articles/PMC11125840/#B18-vaccines-12-00462. 10.3390/vaccines12050462 PMC11125840 38793713

[42] Dameche K, Shams S, AlMesallam MS. Herpes Zoster (HZ) over the past 10 years: a systematic review on trends, triggers, and post-COVID-19 impact. Cureus. 2025;17(12):e98556. Available from: URL: https://pubmed.ncbi.nlm.nih.gov/41503324/. 10.7759/cureus.98556 PMC12770792 41503324

[43] Grandt D, Lappe V, Schubert IBARMER-A. 2025: Herpes zoster – Analysen zu Impfungen und Erkrankungen; September 2025 [cited 2026 Apr 4]. Available from: URL: https://www.barmer.de/resource/blob/1387224/6b58f6b9642c6be1637fbb5d7c3a81f7/dl-arzneimittelreport-2025-data.pdf.

[44] Gemeinsamer Bundesausschuss. Impfung gegen Gürtelrose wird Kassenleistung; 2026 [cited 2026 Jun 25]. Available from: URL: https://www.g-ba.de/presse/pressemitteilungen-meldungen/786/.

