# Supplementary material for: Clinical and epidemiological characteristics of herpes zoster at a tertiary care center compared to population-based data: a ten-year retrospective cohort study
Source: BMC Infect Dis. 2026 Aug 31;26:1596. doi: 10.1186/s12879-026-14185-7 (PMC13536859; doi:10.1186/s12879-026-14185-7)
Supplement: Supplementary file 1 [file 12879_2026_14185_MOESM1_ESM.docx]

no. of cases

| year of Diagnosis | Total cases | Hospitalised cases | PHN cases |
| --- | --- | --- | --- |
| 2014 | 153 | 116 | 12 |
| 2015 | 77 | 44 | 11 |
| 2016 | 48 | 27 | 6 |
| 2017 | 35 | 17 | 2 |
| 2018 | 23 | 17 | 2 |
| 2019 | 39 | 21 | 2 |
| 2020 | 47 | 26 | 5 |
| 2021 | 83 | 31 | 6 |
| 2022 | 91 | 42 | 9 |
| 2023 | 190 | 112 | 11 |

**Fig. 1** Annual distribution of herpes zoster cases, hospitalized cases, and postherpetic neuralgia (PHN) at the University Hospital Regensburg, 2014 – 2023
