# Supplementary material for: Clinical and epidemiological characteristics of herpes zoster at a tertiary care center compared to population-based data: a ten-year retrospective cohort study
Source: BMC Infect Dis. 2026 Aug 31;26:1596. doi: 10.1186/s12879-026-14185-7 (PMC13536859; doi:10.1186/s12879-026-14185-7)
Supplement: Supplementary file 2 [file 12879_2026_14185_MOESM2_ESM.docx]

No. of cases

| Month of diagnosis | Total cases |
| --- | --- |
| 1 | 73 |
| 2 | 60 |
| 3 | 55 |
| 4 | 48 |
| 5 | 64 |
| 6 | 85 |
| 7 | 64 |
| 8 | 76 |
| 9 | 67 |
| 10 | 52 |
| 11 | 61 |
| 12 | 81 |

**Fig. 2** Monthly distribution of herpes zoster cases at the University Hospital Regensburg, 2014 – 2023
