# Supplementary material for: Clinical and epidemiological characteristics of herpes zoster at a tertiary care center compared to population-based data: a ten-year retrospective cohort study
Source: BMC Infect Dis. 2026 Aug 31;26:1596. doi: 10.1186/s12879-026-14185-7 (PMC13536859; doi:10.1186/s12879-026-14185-7)
Supplement: Supplementary file 3 [file 12879_2026_14185_MOESM3_ESM.docx]

**Table 1.** Available data regarding SARS-CoV-2

| **Available data on SARS-CoV-2^a^** | **Total (n = 49)** |
| --- | --- |
|  | **n** |
| Vaccinated | 6 |
| Current infection | 7 |
| Current COVID – Pneumonia^b^ | 6 |
| Current COVID – ARDS^c^ | 4 |
| COVID history | 5 |
| COVID – pneumonia history | 1 |
| Negative test result | 29 |

Number of cases (n)

^a^ Severe Acute Respiratory Syndrome Coronavirus 2

^b^ Coronavirus Disease 2019 Pneumonia

^c^ Coronavirus Disease 2019 – associated Acute Respiratory Distress Syndrome
